# Exposure to Pollutants and Vaccines’ Effectiveness: A Systematic Review

**DOI:** 10.3390/vaccines12111252

**Published:** 2024-11-03

**Authors:** Carmela Protano, Federica Valeriani, Katia Vitale, Jole Del Prete, Fabrizio Liguori, Giorgio Liguori, Francesca Gallè

**Affiliations:** 1 Department of Public Health and Infectious Diseases, Sapienza University of Rome, 00185 Rome, Italy; carmela.protano@uniroma1.it (C.P.); katia.vitale@uniroma1.it (K.V.); jole.delprete@uniroma1.it (J.D.P.); 2 Department of Movement, Human and Health Sciences, University of Rome Foro Italico, 00135 Rome, Italy; federica.valeriani@uniroma4.it; 3 Department of Economics and Legal Studies, University of Naples “Parthenope”, Via Generale Parisi 13, 80132 Naples, Italy; fabrizio.liguori@studenti.uniparthenope.it; 4 Department of Medical, Movement and Wellbeing Sciences, University of Naples “Parthenope”, 80133 Naples, Italy; giorgio.liguori@uniparthenope.it

**Keywords:** environmental pollution, toxics, human health, infectious disease prevention, immunization, vaccine, systematic review

## Abstract

**Background:** Many human activities release harmful substances, contaminating the air, water, and soil. Since exposure to environmental pollutants is currently unavoidable, it is important to verify how these compounds may influence individual immune responses to vaccines. **Methods:** This review was conducted in accordance with the PRISMA statement. The protocol was registered on the PROSPERO platform with the following ID: CRD42024582592. We evaluated all observational, semi-experimental, and experimental studies written in both Italian and English that reported possible effects of exposure to environmental pollutants on the production of vaccine-induced antibodies. **Results:** Forty-two studies were included. The effects of pollutants were examined mainly in terms of antibody production in relation to mumps, measles and rubella, diphtheria and tetanus, hepatitis A and B, Haemophilus influenzae type B, influenza, tuberculosis, pertussis, Japanese encephalitis, poliomyelitis, and COVID-19 vaccines. Perfluorinated compounds were the most studied pollutants. **Conclusions:** Correlations between exposure to pollutants and reductions in antibody production were found in quite all the selected studies, suggesting that pollution control policies could contribute to increase the efficacy of vaccination campaigns. However, the heterogeneity of the examined studies did not allow us to perform a meta-analysis, and the literature on each type of vaccine or pollutant is still too limited to generate robust evidence. In order to confirm the findings of the present systematic review, and in the perspective of establishing possible exposure limit values for each type of pollutant, further research in this field is required.

## 1. Introduction

Being the most efficient way to stop the spread, morbidity, and mortality of several infectious diseases, vaccination is considered one of the most important public health achievements of the 20th century [1]. The World Health Organization (WHO) estimates that since 1974, vaccination has prevented 154 million deaths worldwide [2]. This practice consists of a process of active immunization: a healthy subject is exposed to a specific antigen, inducing the development of an adaptive immune response to that antigen [3]. However, numerous variables endogenous (genetics, age, and sex) and exogenous (stress, alcohol, physical activity, diet, and infectious illnesses) may influence the effectiveness and duration of vaccine-induced protection [4].

Environmental pollution affects the entire world population and it is a growing problem. In fact, many human activities release substances harmful to health into the environment, contaminating the main environmental matrices in contact with humans: air [5], water [6], and soil [7]. As scientific and technological progress advances and anthropic activity increases, pollution has begun to spread more and more [8] and it has become a serious public health problem [9]. According to the Lancet Commission on Pollution and Health, which used data from the Global Burden of Disease, Injury and Risk Factors 2019, pollution was responsible for 9 million deaths a year, about one in six deaths worldwide, making it the biggest environmental risk factor for disease and premature death [10]. According to WHO data, more than 99% of the global population breathes air where pollution is greater than the levels indicated by the WHO air quality guidelines, and 4.2 million deaths each year are associated with air pollution [11].

Many kinds of environmental pollutants were studied in order to evaluate the impact of exposure to them on human health: for example, an association is reported in the literature between particulates and cardiovascular diseases [12], but also for other pathologies such as chronic kidney disease [13], impaired cognitive function, preterm births, allergies [14], and diabetes [15], between heavy metals and metabolic diseases such as osteopenia and osteoporosis [16], between pesticides and respiratory system diseases like asthma and infections [17], and between per- and polyfluoroalkyl substances (PFASs) and dysregulation of thyroid function [18]. Pollutants such as particulate matter and polycyclic aromatic hydrocarbons have also been shown to be involved in carcinogenesis processes [19,20].

Since pollution exposure is currently unavoidable, it is reasonable to wonder how these compounds can affect individual immune responses to vaccines, which are likely the most commonly used public health tool worldwide. Therefore, this systematic review aims to provide an overview of the scientific data regarding the potential effects of exposure to environmental pollutants on vaccine efficacy, evaluated in terms of the individual production of vaccine-induced antibodies.

## 2. Materials and Methods

### 2.1. Research Strategy

This review was conducted in accordance with the PRISMA (Preferred Reporting Items for Systematic Reviews and Meta-Analyses) Statement [21]. The protocol was registered on the PROSPERO platform with the following ID: CRD42024582592. The protocol is available from: https://www.crd.york.ac.uk/prospero/display_record.php?ID=CRD42024582592 (accessed on 27 October 2024).

The bibliographic and citation databases PubMed (Medline), Scopus, and Web of Science (Science and Social Science Citation Index) were examined. The following keywords with Boolean operators as AND–OR were used for the query: (“environmental pollution” [MeSH] OR “water pollution” [MeSH] OR “indoor pollution” OR “outdoor pollution” OR “water pollutant*” OR “soil pollutant*” OR “air pollutant*” OR “indoor pollutant*” OR “outdoor pollutant*” OR “environmental toxicants exposure” OR “environmental pollutants exposure” OR “environmental toxicants” OR “environmental pollutants”) AND (“vaccine*” OR “vaccination*” OR “vaccine efficacy” OR “vaccination efficacy” OR “immunization” OR “antibody response to vaccination” OR “humoral immunity” OR “vaccine antibody levels”). The search was conducted from 9 August 2024 to 30 August 2024 All articles published from the inception until 30 August 2024 were included in the research.

### 2.2. Inclusion and Exclusion Criteria

We evaluated studies written in both Italian and English language which reported possible effects of exposure to environmental pollutants on immune responses to vaccination. Studies that reported no original data, including reviews, systematic reviews, case studies, proceedings, qualitative investigations, book chapters, editorials, and opinion articles, were omitted. All observational, semi-experimental, and experimental studies on humans reporting original data on the studied issue were included. In addition, we performed citation chaining, examining the reference lists of the recovered studies in order to identify further articles on the focus of the present systematic review. Any article that did not meet the inclusion criteria was excluded. The PICOS model was employed for structuring the research question as follows:

Population: all people (individuals of all gender, age, ethnicity and health conditions) vaccinated against any vaccine-preventable disease.
Intervention: exposure to environmental pollutants.
Control: age-, gender- and condition-matched not vaccinated or vaccinated but differently exposed to pollutant(s).
Outcomes: effects of exposure to environmental pollutants on vaccine-induced immune response, assessed through vaccine-induced antibody levels.
Study: observational studies and semi-experimental and experimental studies on humans.

All studies that did not satisfy the inclusion criteria were excluded.

The references of the chosen articles were all transferred to the Zotero citation management software (RRID:SCR_013784, version 6.0.36) in order to evaluate the significance of each article and eliminate any duplicates.

Initially, titles and abstracts were evaluated by three researchers (J.D.P., K.V. and F.L.) to independently verify the potentially qualifying papers. Three content experts (C.P., F.G., and F.V.) helped with the reviewing and evaluating process. Subsequently, two researchers (J.D.P. and K.V.) assessed the full text of each included article independently. The group discussed and resolved any disagreements on the chosen papers.

### 2.3. Risk of Bias Assessment

Forty-one observational studies were obtained at the end of the evaluation and selection process.

The quality of the observational studies was evaluated using the Newcastle–Ottawa scale (NOS) modified for cohort, case-control, and cross-sectional studies. This scale was used to compute the total rating. Each study was reviewed separately by two authors (J.D.P. and K.V.) and disagreements were resolved by the authors debating with one another. The final rating for each article was determined by taking the mean value of the authors’ scores. The scale has different numbers of questions and scores for different studies: for case-control and cohort studies, 8 questions for a maximum score of 9, and for cross-sectional studies, 6 questions with a maximum score of 7. In particular, the three categories assessed were selection, comparability, and outcome, and a score was given to each based on the type of study: for cross-sectional studies, 3 points for selection, 2 for comparability, and 2 for outcome, with a maximum total score of 7; and for case-control and cohort studies, 4 points for selection, 2 for comparability, and 3 for outcome, with a maximum total score of 9. A score of 7 to 9 points denotes good quality (low risk of bias), 5 to 6 points denotes fair quality (moderate risk of bias), and 0 to 4 points denotes poor quality (high risk of bias) [22].

The quality assessment was entered into the data extraction table. Additionally, the following details were listed in this table for each article: author, publication year, country, presence of a sponsor, study design, sample size and main population characteristics, vaccination features, type of pollutant(s), exposure assessment, main findings, and quality of the included studies.

## 3. Results

**Figure 1:**
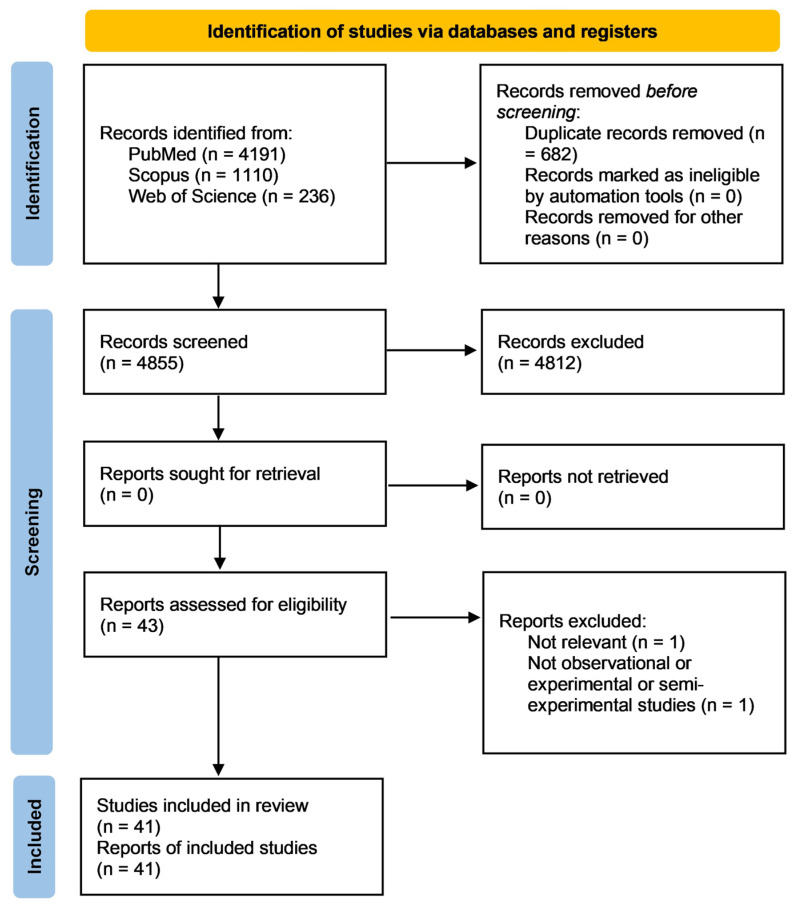
Prisma flow chart describing the research strategy.

Figure 1 shows the flow chart of the review process.

Using the search query, 5537 records were found in the three databases: PubMed (4191), Scopus (1110), and Web of Science (236). After removing the duplicates (682 studies), the title and abstract of 4855 articles were evaluated and the full texts of 43 records were screened. Out of these, two were eliminated due to non-compliance with the eligibility requirements. Upon ending the process, the systematic review included 41 articles.

The data extracted from the included studies are summarized in the Supplementary Table S1 (characteristics of the included studies) and in Table 1 (main results and results of the quality assessment of the included studies).

Eight of the 41 articles included in the present review reported cross-sectional studies [28,33,37,42,46,50,56,61], while the others reported cohort studies [23,24,25,26,27,29,30,31,32,34,35,36,38,39,40,41,43,44,45,47,48,49,51,52,53,54,55,57,58,59,60,62,64].

Most of the articles included studies carried out in the United States [24,31,33,37,38,41,43,46,47,51,53,54,57,58,59,62], followed by Denmark [26,29,36,39,43,49,55,63], and China [30,35,42,56,61].

Heavy metals, polychlorinated biphenyls (PCBs), and per- and polyfluoroalkyl substances (PFASs) were the most studied pollutants. To a lesser extent, the included studies evaluated the effect of pesticides [27,28,38,53,57], particulate matter [56,60], and phthalates [52]. The most common method used for assessing exposure to studied pollutants was human biomonitoring using serum or blood samples, with the exception of one study [56] that evaluated the exposure to environmental pollution through monitoring stations of air pollutant concentrations.

Most of the included studies evaluated the exposure to environmental pollution of the general population, while occupational exposure was the subject of just three studies: two evaluated the influence of pesticides [27,28] and did not show statistically significant relationships with hepatitis B vaccination, while the third assessed the role of PFAS on COVID-19 vaccination [58] and showed a significant inverse association between exposure to PFAS and the reduction of vaccine efficacy.

The majority of the vaccinations under investigation were hepatitis B, rubella, measles, mumps, diphtheria, and tetanus [23,33,35,36,39,40,42,43,44,45,46,47,48,49,50,51,52,53,54,55,57,61,62,63]. In addition, a smaller number of studies considered vaccines against Haemophilus influenzae type B (HiB) [30,32,47,48,50], influenza [34,41], Bacillus Calmette-Guérin (BCG) [38], hepatitis type A [37,54], Japanese encephalitis [42], polio [42], and COVID-19 [56,58,59,60] vaccinations.

Many articles included in this review showed evidence of the interference that different classes of environmental pollutants exert on vaccine efficacy [25,26,29,31,32,33,34,35,36,38,40,42,43,44,45,46,47,48,49,50,51,52,53,56,57,58,60,61,62,63]. Some research has shown how PFAS exposure can negatively influence the antibody titers related to influenza vaccines containing the A/H3N2 strain [34], diphtheria and tetanus vaccines [31,36,39], the rubella vaccine in adults, [46] and, overall, the measles, mumps, and rubella (MMR) vaccine and the diphtheria, tetanus, and pertussis (DTP) vaccine [63]. However, conflicting results emerged: in one study, a statistically significant decrease emerged only for diphtheria but not for tetanus [44]; in another, the decrease in antibody titers related to measles was evident at a first follow up performed on 9-month-old children but not in a second one at the age of 2 years [49]. In other studies, only some PFASs (e.g., perfluorooctanoic acid, PFOA) [43] showed negative associations with diphtheria, tetanus, and HiB antibody titers while others did not (e.g., perfluorooctane sulfonate, PFOS) [50]. In another investigation, increased exposure to PFASs led to a decrease in rubella and mumps antibodies in the same participants and, at the same time, an increase in measles antibodies [61]. However, other studies which analyzed the role of PFASs did not yield any statistically significant results [41,54,55,59].

Additionally, a lot of research has been done on heavy metals, which are ubiquitous and have been shown to have a negative effect on vaccine-induced antibody concentration [42]. Lead exposure has been shown in various studies to lower the antibody titer of the vaccinations under investigation: tetanus [48] and measles, mumps, and rubella [40]; conversely, in another, there was a rise in the titers of the tetanus antibodies [51]. In one case, it was shown that a reduction in lead exposure resulted in an increase in the titers of hepatitis B antibodies [35]. With regards to mercury, highly variable results emerged: it was shown that as exposure increased, antibody titers for pertussis, diphtheria, and measles decreased, but in the same children with a better nutritional status, a smaller decrease was registered [47]. A similar situation can be seen in another paper on children who had high levels of methylmalonic acid, low folate, and high homocysteine: when mercury exposure increased by one percentage point, an increase in rubella antibody titer of 0.24% emerged, while in the absence of the previously listed factors a 0.18% decrease was found [33]. The results for arsenic, which is a metalloid, are also quite patchy: increases in exposure lead to increases in hepatitis A antibodies [37], a decrease in mumps antibodies [45], and in measles antibodies [62]. Finally, one study produced no significant results [24].

With regard to PCBs, an inverse relationship between exposure and immune response was reported with regard to rubella and mumps [25], diphtheria and tetanus [26,29], and measles [32]. Other studies, however, have not shown statistically significant associations [23,30].

Regarding pesticide exposure, some research has shown a reduction in BCG vaccine antibody levels for tuberculosis [38], tetanus, and diphtheria [57] associated to the increasing levels of pesticides. Further, in one study, a direct proportionality between pesticide exposure and measles antibody titer was found [53], while in another no significance was found [28].

In addition, the effect of particulate matter exposure on COVID-19 vaccination was evaluated and the studies in field reported possible negative interactions [56,60].

Considering the group of phthalates, the contribution of the exposure to these substances was evaluate in one article, which showed a reduction in the antibody titer related to the hepatitis B vaccine [52].

According to the NOS scale, 33 studies presented a good quality [23,24,25,26,27,29,30,31,32,33,34,35,36,38,40,43,44,45,46,47,48,49,51,52,53,54,55,57,58,59,60,61,62,63], seven a fair quality [28,39,41,42,50,56,61], and only one had a poor quality [37].

## 4. Discussion

The aim of this review was to assess the effects of environmental pollution on human immune responses to vaccination. The available literature suggests that the exposure to pollutants may negatively affect the effectiveness of vaccines in terms of antibody production. In particular, 16 studies aimed to address the relationship between PFC compounds and human responses to vaccination [31,34,36,39,41,43,44,46,49,50,54,55,58,59,61,63]. Among these, only the study by Hollister et al. did not report negative effects of exposure on antibody titers [59]. Due to their hydrophobic and oleophobic characteristics, perfluorinated compounds are largely used by humans, especially as fabric and container components, and, consequently, these compounds enter in the environmental matrices and humans are exposed to them. In particular, they can be easily spread in the air, soil, and water, reaching human tissues through the food chain and leading to negative health outcomes. Indeed, scientific evidence in this field has showed that they can affect the liver, central and peripheral nervous system, and endocrine system [64]. In the last few years, the immunotoxicity of PFCs has gained growing attention. Recently, the mechanism by which PFCs bind IgG has been revealed at the molecular level, providing a theoretical basis for their immunotoxic effect [65]. This research might also contribute to understanding the role of PFCs in lowering antibody titers after vaccination.

Eleven of the examined studies were focused on the possible effects of heavy metals [24,33,35,37,40,42,45,46,47,48,51,62]. Heavy metals are metals and metalloids with an atomic density higher than 4000 kg/m^3^ and are toxic to human beings even at low concentrations [66]. Individuals exposed to high amounts of heavy metals may develop gastrointestinal, renal, and cardiovascular diseases, tumors, and osteoporosis. Heavy metals can contaminate soil and water sources through industrial discharges and agricultural runoff. Many countries have established regulations for heavy metals that limit their concentrations in food to reduce their consumption. Significant but not always consistent evidence is available regarding the effects of heavy metals on human immune function, with some studies reporting immunostimulation and consequent hypersensitivity, allergies, and autoimmunity development, and others reporting immunosuppression and increased infection and cancer risk [67]. Our findings may be in line with the latter results. Indeed, all of the included studies showed that these pollutants may alter the immune response to vaccines. Among these, the only study involving adult individuals was the only one reporting higher antibody titers related to exposure to arsenic [37]. Interestingly, two of these studies reported worse effects of exposure to mercury in children who showed nutritional susceptibility, suggesting that nutrition could mediate the interaction between this metal and the immune response [33,47].

Furthermore, six out of eight studies investigating the effects of children’s or maternal exposure to PCBs [25,26,29,32,38,55] reported significant reductions in children’s immune responses to vaccines. PCBs can reach humans through the ingestion of contaminated foods, inhalation of contaminated air, and even dust ingestion or dermal contact. Environmental and occupational exposure to high concentrations of PCBs has been associated with several adverse outcomes, such as cardiovascular diseases and cancers, neurological deficits, dementia, and immune system dysfunctions. In addition, the bioaccumulation of PCBs can reduce fertility, with effects on the reproductive system that can be passed to offspring. Our finding highlights the need of further investigating the effects of prolonged exposure to low concentrations of PCBs [68].

The effects of pesticides were explored by four studies [27,28,53,57]. Two of these studies were performed on adults occupationally exposed and reported no differences in antibody titers between exposed and non-exposed individuals [27,28]. However, Baranska et al. reported that exposed workers genetically characterized by interleukin-1 gene polymorphism presented a lower immune response, demonstrating the relevance of the genetic variability in the evaluation of the effects determined by environmental exposure and the possibility of high-risk individuals [28]. Prahl et al. [53] and Hammel et al. [57] focalized their attention on children exposed to pesticides and their immune responses, reporting, respectively, higher and lower antibody levels in exposed children. The difference between age classes, which was also reported by Pilkerton et al. for PFCs [46], deserves attention in light of the definition of limit values for exposure to such compounds for individuals of different ages. Moreover, as also reported by Cardenas et al. [37], for arsenic, some pollutants may exert immune dysregulation, which can lead to the detection of increased antibody levels. This aspect should be considered when interpreting the results of similar studies.

The studies by Zhang et al. and Kogevinas et al. analyzed the interaction between air pollutants and immune responses to vaccines [56,60] and both of them showed an inverse relationship between exposure to pollution and anti-vaccine Ig levels. Finally, Wen et al. assessed and verified the negative effects of phthalates on vaccine effectiveness.

Taken together, all this evidence highlights the negative effects of environmental pollution on the immune system and suggests that it can also reduce the effectiveness of active immunization. Besides the technical, economic, political, and demographic issues that can reduce the effectiveness of vaccination programs [69], this aspect can contribute to hindering the control of vaccine-preventable infectious diseases and should be considered by health institutions.

This review shows important limitations. First of all, most of the findings have come from the US and the Netherlands. This result is due to the fact that all the studies performed in this field and published in international journals in the English language were carried out in those countries. We selected only studies in English because most peer-reviewed articles are published in this language, In addition, we also selected articles published in Italian, the language of the authors, but no articles in Italian were included in this review. Previous systematic reviews [70,71] demonstrated that restricting the search strategy to publications in the English language has little influence on the conclusions of systematic reviews on conventional and alternative medicine. To the contrary, other research has highlighted that studies published in languages different from English frequently present methodological limitations [72]. Although the majority of the included studies showed a good quality and reported the same measure of vaccine effectiveness and also the same type of pollutant exposure assessment, it should be considered that they examined the responses to different type of vaccines and the effects of different pollutants. Therefore, our findings should be considered with caution. Nevertheless, the literature regarding each type of vaccine or pollutant is still too scarce to generate robust evidence. In order to confirm our findings, and in the perspective of establishing possible exposure limit values for each type of pollutant, further research in this field is required. The systematic analysis of comparable studies, selected through more specific eligibility criteria, would allow to formulate stronger conclusions.

## 5. Conclusions

The results of the present systematic review showed that, overall, environmental pollution can negatively influence the human immune response to vaccines. This evidence is of great importance for public health because vaccines are one of the most relevant instruments available to prevent and control infectious diseases. The increasing environmental issues represent a multifaceted threat for population health. In this perspective, pollution control policies assume a fundamental role since they can also contribute to increase the effectiveness of immunization campaigns. However, the examined studies showed a great heterogeneity due to the variability in vaccines and pollutants investigated, but also in the type of populations examined and in immune response indicators evaluated. Furthermore, the individual differences in immune response should be taken in account when considering these data. Thus, it is essential to perform further studies to define the effects of environmental pollution on vaccines effectiveness. In particular, controlled studies focused on specific types of vaccines and pollutants and using the same assessment methods to evaluate immune response in comparable population groups are needed to obtain comparable results.

## Figures and Tables

**Table 1:** Main results and quality assessment of the included studies.

Author, Year, Country, Sponsor	Main Results	Quality Assessment
Weisglas-Kuperus et al. [23], 1995, Netherlands	No significant correlations between mumps, measles, and rubella antibodies and pre-/postnatal PCB/dioxin exposure.	Good
Lutz et al. [24], 1999, USA	A statistically significant relationship between IgE and blood lead level. Statistically non-significant negative association between Rubella index in serum and blood lead values (correlation coefficient 0.09, *p* < 0.15).	Good
Weisglas-Kuperus et al. [25], 2000, Netherlands	Negative correlation between mumps antibodies and PCB maternal levels (r = −0.17, *p* = 0.04) and between rubella antibodies and PCB cord levels (r = −0.19, *p* = 0.03).	Good
Heilmann et al. [26], 2006, Denmark	Decrease in diphtheria antibodies at 18 months by 24.4% (*p* = 0.04) for each doubling of cumulative PCB exposure. At 7 years, lower diphtheria response, not associated with the exposure. Tetanus toxoid antibodies affected mainly at 7 years, decreased by 16.5% (*p* = 0.03) for each doubling of prenatal exposure.	Good
Steerenberg et al. [27], 2008, Netherlands	No differences in HBV antibodies at day 49 after the first vaccination. Similar levels of HBV IgG in exposed (mean ± SD, 2.1 ± 1.9 IU/L) and non-exposed (mean ± SD, 2.3 ± 2.0 IU/L).	Good
Baranska et al. [28], 2008, Poland	No statistically significant differences in HBV antibodies (exposed and control groups: 2.28 ± 2.01 vs. 2.12 ± 1.85).	Fair
Heilmann et al. [29], 2010, Denmark	Relative change in serum tetanus antibody concentrations associated with a doubling of PCB exposure during development: statistically non-significant reduction of 6% at age 5 years and reduction of 21.7% (*p* < 0.003) at age 7 years. Relative change in serum diphtheria antibody concentrations associated with a doubling of PCB exposure during development: 15.4% reduction (*p* < 0.01) at age 5 years and 18.3% reduction (*p* < 0.03) at age 7 years.	Good
Jusko et al. [30], 2010, USA	No significant association between pre- or postnatal PCB levels and antibody levels at 6 months of age.	Good
Grandjean et al. [31], 2012, USA	A 2-fold increase in PFOS exposure associated with a −39% (95% CI, −55% to −17%) difference in antibody levels at 5 years before the booster. At 7 years, 2-fold increase in PFOA exposure associated with differences of −36% (95% CI, −52% to −14%) and −25% (95% CI, −43% to −2%) for tetanus and diphtheria, respectively. PFOS exposure associated with a difference in diphtheria antibody levels of −28% (95% CI, −46% to −3%).	Good
Stølevik et al. [32], 2013, Norway	Maternal exposure to dioxins and dl-PCBs or ndl-PCBs associated with reduced levels of measles antibodies (*p* = 0.032, *p* = 0.036). No significant associations between antibody levels from other vaccines and exposure to dioxins and dl-PCBs or ndl-PCBs.	Good
Gallagher et al. [33], 2013, USA	Exposure to mercury significantly positively associated with rubella antibody levels (0.24% increase per 1% increase of blood mercury; β = 0.24; 95% CI = 0.11, 0.38) in children with nutritional susceptibility (higher methylmalonic acid, lower folate, and higher homocysteine) and inversely associated in others (0.18% decrease per 1% increase of blood mercury; β = −0.18; 95% CI = −0.34, −0.03).	Good
Looker et al. [34], 2014, UK, Supported by C8 Class Action Settlement Agreement DuPont—Plaintiffs.	A/H3N2 antibody levels were negatively associated with PFOA concentrations (adjusted coefficient −0.12 [95% CI: −0.25, 0.02, *p* = 0.009]). Largest reduction in A/H3N2 antibody levels resulted for the highest quartile of concentrations of PFOA (adjusted coefficient −0.22 [95% CI: −0.43, −0.01]).	Good
Xu et al. [35], 2015, China	For the 2011 group: higher blood levels of Pb and lower titers of HBsAb in were found in exposed children respect to the reference group (8.76 µg/dL vs. 7.89 µg/dL; 0.83 s/co vs. 4.64 s/co, respectively). For the 2012 group: higher blood levels of Pb and lower titers of HBsAb were recovered in exposed children compared to the reference group, (5.83 µg/dL vs. 4.61 µg/dL and 1.31 s/co vs. 3.80 s/co, respectively).	Good
Mogensen et al. [36], 2015, Denmark	Higher PFAS concentrations measured after 7 years were associated with lower levels of antibodies. Diphtheria antibody levels decreased respectively by 30.3% (95% CI: 7.8%, 47.3%) and 25.4% (95% CI: 5.8%, 40.9%) for double concentration of PFOS and PFOA. Tetanus antibodies decreased by 22.3% (95% CI: 5.2%, 36.3%) for double concentration of PFHxS.	Good
Cardenas et al. [37], 2016, USA	HAV antibodies associated with As levels, in relationship with immunization status (P = 0.03). In participants that received ≥ 2 vaccine doses or did not know if they had received any doses, positive dose-response association with anti-HAV (OR 1.75, 95% CI: 1.22–2.2); in those who received < 2 doses or no dose, positive but not statistically significant association (OR 1.46, 95% CI: 0.83–2.59 and OR 1.12, 95% CI: 0.98–1.30, respectively).	Poor
Jusko et al. [38], 2016, USA	Higher PCB-153 and DDE concentrations were strongly associated with lower antibody levels specific for BCG: IgG levels specific for BCG were 37% lower for infants with PCB-153 concentrations at the 75th percentile compared to the 25th percentile (95% CI: −42, −32; *p* < 0.001). Similar findings were recovered for DDE. The exposure to both pollutants more reduced anti-BCG.	Good
Kielsen et al. [39], 2016, Denmark	Antibody levels negatively affected by the majority of PFASs. Diphtheria antibodies titer/exposure to PFHxS −13.3% *p* < 0.05. PFOS −11.9% *p* < 0.04. PFNA −17.9% *p* < 0.004. PFDA −18.2% *p* < 0.009. PFUnDA −12.1% *p* < 0.03. PFDoDA −15.64% *p* < 0.03. Tetanus antibodies titer/exposure to PFUnDA −7.9% *p* < 0.03. PFDoDA −10.8% *p* < 0.03.	Fair
Lin et al. [40], 2016, China	Median titer of measles IgG of exposed subjects decreased of about 40% (669.64 mIU/mL, IQR 372.88–1068.42 mIU/mL) with respect to the reference individuals (median 1046.79 mIU/mL, interquartile range 603.29–1733.10 mIU/mL). The mumps IgG levels decreased by about 45% in the exposed group (median = 272.24 U/mL, IQR = 95.19–590.16 U/mL) with respect to the reference group (median = 491.78 U/mL, IQR = 183.38–945.96 U/mL). Rubella antibodies decreased by about 44% in the exposed group compared to the reference. Anti-rubella antibody levels were below the protective level in 26% of the exposed subjects and in 15% of the reference individuals.	Good
Stein et al. [41], 2016, USA	PFOS, PFOA, PFHxS, or PFNA concentrations were not associated with antibody levels. PFOS, PFOA, PFHxS, or PFNA tertile concentrations were not associated with increasing or decreasing levels of immune markers.	Fair
Lin et al. [42], 2017, China	The OR of blood concentrations of Zn for diphtheria antibody levels was equal to 0.477 (*p* = 0.002). The OR for high levels of blood Pb (Pb levels equal to 5–10 μg/dL) and pertussis and diphtheria levels of antibodies was, respectively, 0.361 (95% CI: 0.160–0.818, *p* = 0.015) and 0.54 (95% CI: 0.247–1.180, *p* = 0.05). The ORs for higher blood levels of Pb (>10 μg/dL) and levels antibodies were in the range of 0.31–0.454 (diphtheria *p* = 0.031, pertussis *p* = 0.01, tetanus *p* = 0.058, hepatitis B *p* = 0.025, Japanese encephalitis *p* = 0.041, polio *p* = 0.04, and measles *p* = 0.08). The ORs for high blood concentrations of Cu and the levels of antibodies were in the range of 0.471-0.598 (diphtheria *p* = 0.081, pertussis *p* = 0.07, tetanus *p* = 0.049, hepatitis B *p* = 0.097, and Japanese encephalitis *p* = 0.014). The ORs for high blood concentrations of Zn and antibody levels were in the range of 0.483–0.563 (Japanese encephalitis *p*= 0.021, polio *p* = 0.076, and measles *p* = 0.062). High blood concentrations of As, Hg, Se, and Cr were not associated with all seven types of studied antibodies.	Fair
Grandjean et al. [43], 2017, USA	At PFAS exposure increase, reduction in tetanus antibody levels [−9.1% (PFOS, *p* < 0.43), −25.3% (PFOA, *p* < 0.031), −4.4% (PFHxs, *p* < 0.65), −10.3% (PFNA, *p* < 0.21), and −1.75% (PFDA, *p* < 0.83)] and in diphtheria antibody levels [−8.8% (PFNA, *p* < 0.32) and −9% (PFDA, *p* < 0.29%)]. Increases in diphtheria antibody levels were also reported: +17.17% (PFOS, *p* < 0.21), +18.31 (PFOA, *p* < 0.24), and +4.26% (PFHxS, *p* < 0.69).	Good
Grandjean et al. [44], 2017, Denmark/USA	For diphtheria antibodies, there were statistically significant inverse associations with all PFASs (PFOS *p* < 0.002, PFOA *p* < 0.045, PFHxS *p* < 0.042, PFNA *p* < 0.002, and PFDA *p* < 0.001). For tetanus, there were no significant inverse associations. Over 7 years, there was a similar indirect effect for diphtheria; for tetanus, there were statistically significant indirect effects both for PFOA (–24.2; 95% CI: −41.1, −2.4) and for PFHxS (–25.1; 95% CI: −38.9, −8.3).	Good
Raqib et al. [45], 2017, Bangladesh	The levels of IgG against mumps decreased with the increasing of urinary concentrations of As at 4.5 and 9 years of age (β = −0.16; 95% CI: −0.33, 0.01; *p* = 0.064 and β = −0.12; 95% CI: −0.27, −0.029; *p* = 0.113, respectively) in 25% of subjects with the lowest preexisting titers of IgG against mumps.	Good
Pilkerton et al. [46], 2018, USA	In subjects aged 12–18 years, rubella antibody levels were not associated with either PFOA or PFOS (PFOA *p* < 0.79 e PFOS *p* < 0.25). In adults, rubella antibodies were significantly associated with both PFOA (*p* = 0.0016) and PFOS (*p* = 0.0295) quartile concentrations.	Good
Wyatt et al. [47], 2019, USA, Supported by Hunt Oil Peru LLC	One-unit increases in hair Hg concentrations in younger children were associated with an increase of IgG respectively equal to 0.68 IU/mL (95% CI: 0.18–1.17) for pertussis and 0.79 IU/mL (95% CI: 0.18–1.70) for diphtheria. Hair Hg concentrations exceeding 1.2 µg/g in older children were associated with 73.7 higher odds (95% CI: 2.7–1984.3) of becoming a non-responder against measles and hair Hg concentrations exceeding 2.0 µg/g with a decrease of IgG equal to 0.32 IU/mL (95% CI: 0.10–0.69) for measles. Older children with poor nutrition presented a reduction of measles antibodies from 1.40 to 0.43 for low exposure (<1.2 µg/g) with respect to high Hg exposure, while children with good nutritional status presented minimal change in measles antibodies for low exposure with respect to high Hg exposure (0.72 vs. 0.81, respectively).	Good
Di Lenardo et al. [48], 2020, Canada	Blood levels of Pb (BLLs) at 1 year were related to vaccine IgG titers at 3.5 years: BLLs were inversely associated with levels of antibodies against tetanus. A 10-fold increase in BLL was associated with a decrease in tetanus IgG eual to 28% (95% CI = −52.16–8.72) and risk of presenting tetanus IgG titers below the protection levels (RR= 1.88; 95% CI = 1.08–3.24; *p* < 0.05). BLLs were not associated with the levels of antibodies against measles or Hib (RR = 1.02; 95% CI = 0.26–3.95, and RR 0.96; 95% CI = 0.54–1.71, respectively).	Good
Timmermann et al. [49], 2020, Denmark	Intervention group: doubling in PFOS and PFDA levels was associated with a decrease of measles antibody titers respectively equal to 21% (95% CI: 2, 37%) and 25% (95% CI: 1, 43%) at the 9-month visit. Control group: elevated PFAS concentrations at inclusion of the study were significantly associated with a reduction in measles levels of antibodies at the 2 y visit (for PFHxS, PFOS, PFOA, and PFNA) only after removal of the most influential points. A doubled concentration of PFOS was associated with a reduction equal to 27% of measles antibody levels (95% CI: 4, 44%).	Good
Abraham et al. [50], 2020, Germany	PFOA levels were significantly associated with Hib (r = −0.32, *p* = 0.001), tetanus IgG1 (r = −0.25, *p* = 0.01), and diphtheria (r = −0.23, *p* = 0.02) antibody levels. No significant associations observed between PFOS and Hib (r = −0.05, *p* = 0.66), tetanus IgG1 (r = −0.07, *p* = 0.52), and diphtheria antibodies (r = −0.02, *p* = 0.84). No significant correlations between PFHxS and PFNA concentrations and vaccine antibody levels.	Fair
Welch et al. [51], 2020, USA	Higher water As during pregnancy was associated with lower concentrations of diphtheria antibodies (−3.4% change per doubling in As, 95% CI: −7.2, 0.6%). Higher blood Pb in pregnancy was associated with higher concentrations of tetanus antibodies (10.2, −0.6%, 22.1%). No associations found between Mn and antibody outcomes.	Good
Wen et al. [52], 2020, Taiwan	Maternal phthalate metabolite levels and percent change in antibody concentrations among children at 11–14 years of age were not significantly associated. Urinary concentrations of MnBP, MEHHP, MEOHP, and ∑DEHPm were associated with a percent change in the decrease of the levels of antibodies against HBV in children aged 11–14 years. Doubled urinary levels of MnBP, MEHHP, MEOHP, and ∑DEHPm were associated with a decrease in HBV antibodies respectively equal to 23% (95% CI = 3.96–41.66%), 18% (95% CI = 3.33–33.38%), 11% (95% CI = 2.77–18.99%), and 18% (95% CI = 3.34–32.78%).	Good
Prahl et al. [53], 2021, USA	Among bendiocarb-exposed infants, a significantly higher anti-measles IgG (*p* < 0.0001) and proportion of infants who were measles IgG-positive following vaccination (91.3% vs. 77.8%, *p* = 0.008) were found.	Good
Shih et al. [54] 2021, USA	Inverse trends between HAV antibodies and PFOA levels were recovered at 14 and 28 years (S/CO change: −0.71; 95% CI: −1.52, 0.09; and S/CO change: −0.24; 95% CI: −0.59, 0.10, respectively). Inverse trends between HBV antibodies and PFOA levels were found at 22 and 28 years (% change: −21.24; 95% CI: −42.20, 7.34; and % change: −16.77; 95% CI: −35.47, 7.35, respectively). Diphtheria antibodies, but not tetanus ones, were positively associated with cord-blood PFAS and PFAS assessed at ages 22 and 28 years.	Good
Timmermann et al. [55], 2022, Denmark	Each 1 ng/mL increase in serum concentration of PFHxS and PFOS was associated with a decrease diphtheria antibody level respectively equal to 78% (95% CI: 25–94%) and 9% (95% CI: 2–16%). Exposure to PCBs and all PFAS concentrations were associated with increased odds of diphtheria antibody levels below the protective level. For each 1 ng/mL increase in serum levels of PFHxS, PFOS, PFNA, and PFDA, an increased odds of not achieving the protective levels of diphtheria antibodies (6.44, 95% CI: 1.51–27.36; 1.14, 95% CI: 1.04–1.26; 1.96, 95% CI: 1.07–3.60; 5.08, 95% CI: 1.32–19.51, respectively) was found. Maternal pollutant concentrations were not consistently associated with the levels of vaccine antibodies.	Good
Zhang et al. [56], 2022, China	Daily exposure dose to air pollutants significantly and negatively associated with plasma-neutralizing antibody titers [B (95% CI): −0.809 (−1.600, −0.019) for PM_2.5_, −0.486 (−0.960, −0.011) for PM_10_, −2.427 (−4.800, −0.055) for SO_2_, −1.139 (−2.211, −0.068) for NO_2_, −0.335 (−0.662, −0.008) for O_3_, −0.034 (−0.067, −0.001) for CO, and −0.485 (−0.954, −0016) for combined toxic effects, all *p* < 0.05].	Fair
Hammel et al. [57], 2022, USA, Supported by Gerber Foundation	In 12-month-old infants, for each increasing of log10-unit of BCIPP levels, there was a decrease of 0.57 IU/mL (95% CI: −1.11, −0.02; *p* = 0.04) in tetanus antibody concentrations. All of the other log10-biomarker concentrations of OPE were associated with an increase of tetanus antibody titers. In 2-month-old infants, each increase in log10-concentration BDCIPP was negatively associated with a decrease of 0.07 IU/mL in diphtheria titers (95% CI: −0.11, −0.03; *p* < 0.001 with of BDCIPP). No other significant associations were found between the levels of OPE metabolites and diphtheria titers.	Good
Porter et al. [58], 2022, USA, Supported by Ramboll and 3M	IgG concentration decreased by about 4% (95% CI −7.03, 0.26) per 14.5 ng/mL (IQR) increase in levels of PFOS. Similar findings were recovered for PFOS concentrations and neutralizing antibodies and for PFOA, PFHxS, and PFNA levels and both neutralizing and IgG antibodies.	Good
Hollister et al. [59], 2023, USA	No statistically significant associations between serum PFAS concentration and antibody levels after vaccination nor between serum PFAS concentration and changes in antibody levels over time.	Good
Kogevinas et al. [60], 2023, Spain	Among participants without prior infection, IgM (within 1 month post first vaccine dose) and IgG levels (any time post vaccination) were negatively associated with long-term air pollution; no associations observed for IgA.	Good
Zhang et al. [61], 2023, China	Serum PFOA levels were negatively associated with rubella antibody titers (PC: −4.36%, 95% CI: −11.53%, 3.40%) and mumps antibodies (PC: −11.05%, 95% CI: −18.56%, −2.85%). PFAS serum levels were not associated with measles antibodies. The quartile increasing in serum levels of the PFAS mixture was associated with an 8% (95% CI: −13.01%, −2.66%) decrease in antibodies for rubella. The quartile increasing in the serum levels of the PFAS mixture was associated with a 5% (95% CI: −10.44%, −0.16%) decrease in antibodies for mumps. Positive joint effects of the PFAS mixture were produced on antibodies for measles (PC: 7.03%, 95% CI: 0.29%, 14.22%).	Fair
Roh et al. [62], 2024, USA	Significant decrease in measles antibody titers (10.8%; *p* < 0.007) for doubled urinary levels of As among individuals with serum folate concentration < 18.7 ng/mL. Stratifying by sex and serum folate levels, a 16.7% decrease in serum measles antibody levels was found (*p* < 0.001) for a doubling of urinary As levels in males with serum folate concentrations < 18.7 ng/mL. In other groups, urinary levels of As were not associated with measles antibody titers.	Good
Sigvaldsen et al. [63], 2024, Denmark	At 18 months, higher serum concentrations of PFAS were associated with lower levels of IgG for measles, mumps, and rubella. Significant differences were found in mumps antibody levels per doubled concentrations of PFNA (−9.2%; 95% CI: −17.4; −0.2), PFHxS (−8.3%; −15.0; −1.0), and PFOS (−7.9%; −14.8; −0.4), but were not significant for PFOA (−5.7%; −12.6; 1.7) and PFDA (−5.6%; −15.6; 5.7). PFAS exposure was inversely associated with tetanus IgG. Higher PFAS concentrations at 18 months of PFHxS concentrations were the only significant association with higher levels of diphtheria IgG.	Good

## Data Availability

No new data were created or analyzed in this study. Data sharing is not applicable to this article.

## Supplementary Materials

The following supporting information can be downloaded at: https://www.mdpi.com/article/10.3390/vaccines12111252/s1, Table S1: Characteristics of the included studies.
