# Supplementary material for: Exposure to Pollutants and Vaccines’ Effectiveness: A Systematic Review
Source: Vaccines (Basel). 2024 Nov 3;12(11):1252. doi: 10.3390/vaccines12111252 (PMC11599004; doi:10.3390/vaccines12111252)
Supplement: Supplementary file 1 [file vaccines-12-01252-s001.zip › vaccines-3267118-supplementary.pdf]

Supplementary file

Table S1. Characteristics of the included studies.

Author, Year, Country, Sponsor	Study Design	Sample size, Study population	Vaccine target	Exposure (pollutant, setting, assessment method)
Weisglas-Kuperus et al. [23], 1995; Netherlands	Cohort study	207 subjects; 46% female; from birth to 18 months of age; caucasian ethnicity	Mumps, measles and rubella	Polychlorinated biphenyls (PCBs) and dioxins<br><br>Environmental exposure<br><br>Biomonitoring using blood samples
Lutz et al. [24], 1999; USA	Cohort study	279 children; 44% females; from 9 months to 6 years of age	Rubella	Lead<br><br>Environmental exposure<br><br>Biomonitoring using blood and serum samples
Weisglas-Kuperus et al. [25], 2000; Netherlands	Cohort study	150 subjects; 42 months of age	Mumps, measles and rubella	Polychlorinated biphenyls (PCBs) and dioxins<br><br>Environmental exposure<br><br>Biomonitoring using serum samples
Heilmann et al. [26], 2006; Denmark	Cohort study	248 children (Group A: 129 children examined at 7 years of age; group B consisted of 119 children examined at 18 months of age)	Diphteria and tetanus	Polychlorinated biphenyls (PCBs)<br><br>Environmental exposure<br><br>Biomonitoring using maternal serum obtained at the 34th week of pregnancy, transition milk, and serum from the child
Steerenberg et al. [27], 2008; Netherlands	Cohort study	479 subjects; 248 exposed workers, 231 non-exposed workers; 33.5% female in exposed workers and 37.6% female in non-exposed workers; average age equal to 42.4 years in exposed workers and equal to 42.2 in non-exposed workers	Hepatitis B	Ethylenbisdithiocarbamate fungicides (EBDC)<br><br>Occupational exposure<br><br>Biomonitoring using urine samples
Baranska et al. [28], 2008; Poland	Cross-sectional study	436 workers: 238 exposed workers and 198 non-exposed workers	Hepatitis B	Pesticides: ethyldithiocarbamates (EBDC)<br><br>Occupational exposure<br><br>Biomonitoring using urine samples
Heilmann et al. [29], 2010; Denmark	Cohort study	587 children; 46.9% Females; age: 7 years	Diphteria and tetanus	Polychlorinated biphenyls (PCBs)<br><br>Environmental exposure<br><br>Biomonitoring using serum samples
Jusko et al. [30], 2010; USA	Cohort study	971 children;49% females; age: from birth to 6 months	Haemophilus influenzae type B, tetanus and diphteria	Polychlorinated biphenyls (PCBs)<br><br>Environmental exposure<br><br>Biomonitoring using maternal, cord an whole blood samples

Grandjean et al. [31], 2012;<br>USA	Cohort study	587 children; 47,4% Females; age: from birth to 7 years	Diphteria and tetanus	Perfluorinated compounds (PFCs): perfluorooctane sulfonic acid (PFOS) and perfluorooctanoic acid (PFOA)<br><br>Environmental exposure<br><br>Biomonitoring using maternal serum samples at the last antenatal examination at week 32 of pregnancy (pre-natal exposure); child of 5 years (post-natal exposure).
Stølevik et al. [32], 2013;<br>Norway	Cohort study	114 subjects; mean age 3.0 years; 63.4% female	Measles, rubella, tetanus and Haemophilus influenzae type b	Dioxins and dioxin-like Polychlorinated biphenyls (PCBs) and non-dioxin-like PCBs maternal intake of the dietary<br><br>Environmental exposure<br><br>Biomonitoring using blood samples
Gallagher et al. [33], 2013;<br>USA	Cross-sectional study	690 children; 52.7% females; age between 6 and 11 years	Rubella	Mercury<br><br>Environmental exposure<br><br>Biomonitoring using blood samples
Looker et al. [34], 2014;<br>UK;<br>Supported by the C8 Class Action Settlement Agreement (Circuit Court of Wood County, WV) between DuPont and Plaintiffs.	Cohort study	411 adults; 48.7% females; age: >18 years	Influenza	Perfluorooctanoate (PFOA) and perfluorooctanesulfonate (PFOS)<br><br>Environmental exposure<br><br>Biomonitoring using serum samples
Xu et al. [35], 2015;<br>China	Cohort study	590 subjects; reference group (289), female (43.25%), average age 4.47 years; exposed group (301), female (45.84%), average age 4.77 years	Hepatitis B	Lead (Pb)<br><br>Environmental exposure<br><br>Biomonitoring using blood samples
Mogensen et al. [36], 2015;<br>Denmark	Cohort study	459 children;48.4% Females; age: 7.5 ± 0.1 years	Tetanus and diphteria	Perfluorinated alkylate substances (PFASs)<br>Perfluorohexane sulfonic acid (PFHxS), perfluorooctane sulfonic acid (PFOS), perfluorooctanoic acid (PFOA)<br><br>Environmental exposure<br><br>Biomonitoring using serum samples

Cardenas et al. [37], 2016, USA	Cross-sectional study	11092 subjects; 50.3% females; age ⩾6 years	Hepatitis A	Arsenic (As)<br><br>Environmental Exposure<br><br>Biomonitoring using urine samples
Jusko et al. [38], 2016; USA	Cohort study	541 children; 50% females; age: from birth to 6 months	Tuberculosis	PCBs (polychlorinated biphenyls) and DDE [1,1-dichloro-2,2-bis(p-chlorophenyl)ethylene]<br><br>Environmental exposure<br><br>Biomonitoring using maternal, cord and whole blood samples
Kielsen et al. [39], 2016; Denmark	Cohort study	12 healthy adult volunteers; 50% females, age: 37.9 (23.2–65.8) years	Tetanus and diphteria	Perfluorinated alkylate substances (PFASs): Perfluorohexane sulfonic acid (PFHxS), perfluorooctane sulfonic acid (PFOS), perfluorooctanoic acid (PFOA), perfluorononanoic acid (PFNA), perfluorodecanoic acid (PFDA), perfluoroundecanoic acid (PFUnDA); perfluoroundecanoic acid (PFUnDA)<br><br>Environmental exposure<br><br>Biomonitoring using serum samples
Lin et al. [40], 2016; China	Cohort study	378; Reference group 115, Female (45.21%), age (4.25 years); Exposed group 263, Female (45.24%), age (4.52 years)	Measles, mumps and rubella	Lead (Pb)<br><br>Environmental exposure<br><br>Biomonitoring using whole blood samples
Stein et al. [41], 2016; USA	Cohort study	78 subjects; mean age 30.2 years; Female (64%); Hispanic ethnicity (22%)	Influenza	Perfluorinated alkylate substances (PFASs): perfluorohexane sulfonic acid (PFHxS), perfluorooctane sulfonic acid (PFOS), perfluorooctanoic acid (PFOA), perfluorononanoic acid (PFNA), perfluorodecanoic acid (PFDA)<br><br>Environmental exposure<br><br>Biomonitoring using serum samples

Lin et al. [42],<br>2017;<br>China	Cross-sectional study	284 children;50% Females; age: from 3 to 7 years	Diphtheria, pertussis, tetanus, hepatitis B, Japanese encephalitis, polio and measles	Arsenic, (As), cadmium (Cd), chromium (Cr), copper (Cu), lead (Pb), manganese (Mn), mercury (Hg), nickel (Ni), selenium (Se), zinc (Zn) ,<br><br>Environmental exposure<br><br>Biomonitoring using whole samples
Grandjean et al. [43],<br>2017;<br>USA	Cohort study	349 children; 49.6% females; age: 5 years	Diphteria and tetanus	Perfluorinated alkylate substances (PFASs): perfluorooctane sulfonic acid (PFOS), perfluorooctanoic acid (PFOA), perfluorononanoic acid (PFNA), perfluorodecanoic acid (PFDA), perfluorohexane sulfonate (PFHxS)<br><br>Environmental exposure<br><br>Biomonitoring using serum samples
Grandjean et al. [44],<br>2017;<br>Denmark/USA	Cohort study	587 subjects;<br>47.4% Females;<br>age: 13 years	Diphteria and tetanus	Perfluorinated alkylate substances (PFASs): perfluorohexane sulfonic acid (PFHxS), perfluorooctane sulfonic acid (PFOS), perfluorooctanoic acid (PFOA), perfluorononanoic acid (PFNA), perfluorodecanoic acid (PFDA)<br><br>Environmental exposure<br><br>Biomonitoring using serum samples
Raqib et al. [45],<br>2017;<br>Bangladesh	Cohort study	525 children 50.8% Females; mean age: 8.9 ± 0.12 years	Measles, mumps and rubella	Arsenic (As)<br><br>Environmental exposure<br><br>Biomonitoring using urine samples
Pilkerton et al. [46],<br>2018;<br>USA	Cross-sectional study	1387 adults<br>41.9% Females<br>age: 34.7 ± 0.7 years;<br>and 1012 youth;<br>age: 15.0 ± 0.6 years	Rubella	Perfluorinated alkylate substances (PFASs): perfluorooctane sulfonic acid (PFOS), perfluorooctanoic acid (PFOA)<br><br>Environmental exposure<br><br>Biomonitoring using serum samples
Wyatt et al. [47],<br>2019;<br>USA;<br>Supported by Hunt Oil Peru LLC	Cohort study	453 subjects; female (52.5%); mean age: <4 (2.4), 4 to 8m (4.5)	Hepatitis B, Haemophilus influenzae type B, measles, pertussis, tetanus and diphtheria	Mercury (Hg)<br><br>Environmental exposure<br><br>Biomonitoring using hair samples
Di Lenardo et al. [48],<br>2020;<br>Canada	Cohort study	406 children vaccinated against measles and 388 vaccinated against tetanus and HiB; 48.7% Females ;mean age 42.9 ±1,20 months	Haemophilus influenzae type B, tetanus and measles	Lead (Pb)<br><br>Environmental exposure<br><br>Biomonitoring using whole blood samples
Timmermann et al. [49],<br>2020;<br>Denmark	Cohort study	194 African children; control group (102): single measles vaccination at 9 months, intervention group (92): two doses of measles vaccine at 4–7 and at 9 months of age<br>Age at inclusion 4.2-7.1 months (mean: 5.6), at	Measles	Perfluorohexane sulfonic acid (PFHxS), perfluorooctane sulfonic acid (PFOS), perfluorooctanoic acid (PFOA), perfluorononanoic acid (PFNA), perfluorodecanoic acid (PFDA), and perfluoroundecanoic acid (PFUnDA)<br><br>Environmental exposure<br><br>Biomonitoring using serum samples

		the 9-month visit 8.9-18.2 months (mean: 9.9), at the 2-y visit 21.9-32.7 months (mean: 25.3).		
Abraham et al. [50], 2020, Germany	Cross-sectional study	101 children, 51 Female;21 formula fed-infants, 11 Females 80 breastfed infants, 39 females mean age: 50,1 weeks (48,7-52,7 weeks)	Diphteria, tetanus and Haemophilus influenzae type B	Perfluorinated alkylate substances (PFASs): perfluorohexane sulfonic acid (PFHxS), perfluorooctane sulfonic acid (PFOS), perfluorooctanoic acid (PFOA), perfluorononanoic acid (PFNA)<br><br>Environmental exposure<br><br>Biomonitoring using plasma samples
Welch et al. [51], 2020; USA	Cohort study	502 subjects; 49% female; from 12 months to 5 years old	Diphtheria and tetanus	Arsenic (As), manganese (Mn) and lead (Pb)<br><br>Environmental exposure<br><br>Environmental monitoring using drinking water for As and Mn<br>Biomonitoring using blood samples for Pb
Wen et al. [52], 2020; Taiwan	Cohort study	81 subjects; 54.3% female; from birth to 15 years old	Diphteria, tetanus, rubella and hepatitis B	Phthalic acid esters (PAEs): monoethyl phthalate (MEP), diethyl phthalate (DEP), monomethyl phthalate (MMP), dimethyl phthalate (DMP), mono-n-butyl phthalate (MnBP), di-n-butyl phthalate (DnBP), monobenzyl phthalate (MBzP), butyl benzyl phthalate (BBzP), mono(2-ethylhexyl) phthalate (MEHP), mono(2-ethyl-5-hydroxyhexyl) phthalate (MEHHP), mono(2-ethyl-5-oxohexyl) phthalate (MEOHP), di-ethylhexyl phthalate (DEHP)<br><br>Environmental exposure<br><br>Biomonitoring using urine samples
Prahl et al. [53], 2021; USA	Cohort study	248 children; age: 1 years	Measles	Pesticides: bendiocarb; Environmental
Shih et al. [54] 2021; USA	Cohort study	1022; 399 (full vaccination course of Twinrix), female (44.9%), 281 (a booster vaccination against diphtheria and tetanus), female (52,7%)	Hepatitis A or B, diphtheria and tetanus	Perfluorinated alkylate substances (PFAS): perfluorooctanoic acid (PFOA), perfluorononanoic acid (PFNA), perfluorodecanoic acid (PFDA), perfluorohexane sulfonic acid (PFHxS), perfluorooctane sulfonate (PFOS)<br><br>Environmental exposure<br><br>Biomonitoring using umbilical cord-blood and serum collected at ages 7, 14, 22, and 28 years (baseline blood sample)
Timmermann et al. [55], 2022; Denmark	Cohort study	338 subjects; Female (48%); median age 9.9 years	Diphtheria and tetanus	Mercury (Hg), polychlorinated biphenyls (PCBs) and perfluorohexane sulfonic acid (PFHxS), perfluoroheptanesulfonic acid (PFHpS), perfluorooctane sulfonic acid (PFOS), perfluorooctanoic acid (PFOA), perfluorononanoic acid (PFNA), perfluorodecanoic acid (PFDA), and perfluoroundecanoic acid (PFUnDA)<br><br>Environmental exposure<br><br>Biomonitoring using serum samples

Zhang et al. [56], 2022; China	Cross-sectional study	207 subjects;66.6% female (66.66%); median age 31 years	COVID-19	Fine particulate matter ($PM_{2.5}$ and PM_{10}), nitrogen dioxide (NO_2), carbon monoxide (CO), sulfur dioxide (SO_2), ozone (O_3)<br><br>Environmental exposure<br><br>Environmental monitoring using stations monitoring air pollutant concentrations
Hammel et al. [57], 2022; USA; Supported by the Gerber Foundation	Cohort study	101 children; 40.6% Females; age: from 2 months to 12 months	Diphteria and tetanus	Organophosphate esters (OPEs)<br><br>Environmental exposure<br><br>Biomonitoring using serum and urine samples
Porter et al. [58], 2022; USA; Supported by Ramboll and 3M	Cohort study	415 adult subjects; 27.2% Females; age: >18 years	COVID-19	Perfluorinated alkylate substances (PFAS): perfluorooctanoic acid (PFOA), perfluorononanoic acid (PFNA), perfluorodecanoic acid (PFDA), perfluorohexane sulfonic acid (PFHxS), perfluorooctane sulfonate (PFOS)<br><br>Occupational exposure<br><br>Biomonitoring using serum samples
Hollister et al. [59], 2023; USA	Cohort study	860 subjects; 67.8% Females; age: 45.5 ± 10.8	COVID-19	Perfluorinated alkylate substances (PFAS)<br><br>Environmental exposure<br><br>Biomonitoring using serum samples
Kogevinas et al. [60], 2023; Spain	Cohort study	927 subjects; 58% Females; age: 57.5 ± 6.9 years	COVID-19	Air pollution: fine particulate matter, nitrogen dioxide, black carbon, and ozone<br><br>Environmental exposure<br><br>Biomonitoring using blood samples
Zhang et al. [61], 2023; China	Cross-sectional study	819 subjects; Female (49.78%); mean age 15.50 years<br>Non-Hispanic White (60.25%)<br>Non-Hispanic Black (14.60%)	Rubella, measles and mumps	Perfluorinated alkylate substances (PFAS): perfluorooctanoic acid (PFOA), perfluorononanoic acid (PFNA), perfluorohexane sulfonic acid (PFHxS) and perfluorooctane sulfonate (PFOS)<br><br>Environmental exposure

		Hispanic (17.88%)<br>Other (7.17%)		Biomonitoring using serum samples
Roh et al. [62],<br>2024;<br>USA	Cohort study	476 children; 45.3% females; age: 8.71 years (95% CI 8.53, -8.89)	Measles	Arsenic (As)<br><br>Environmental exposure<br><br>Biomonioring using urine samples
Sigvaldsen et al. [63],<br>2024;<br>Denmark	Cohort study	880 subjects; 880 children with positive response for DTaP and 841 children with positive response for MMR; 44% female	Measles, mumps, rubella, diphtheria, tetanus and pertussis	Perfluorinated alkylate substances (PFAS): perfluorooctanoic acid (PFOA), perfluorononanoic acid (PFNA), perfluorodecanoic acid (PFDA), perfluorohexane sulfonic acid (PFHxS), perfluorooctane sulfonate (PFOS)<br><br>Environmental exposure<br><br>Biomonitoring using serum samples
